# Unveiling the Mechanisms for the Development of Cardiotoxicity Following Chemotherapy Regimens Administration for Primary Colorectal Cancer: A Systematic Review

**DOI:** 10.3390/cancers17193129

**Published:** 2025-09-26

**Authors:** Sophia Tsokkou, Ioannis Konstantinidis, Paraskevi Chatzikomnitsa, Menelaos Papakonstantinou, Evdokia Toutziari, Dimitrios Giakoustidis, Theodora Papamitsou, Vasileios Papadopoulos, Alexandros Giakoustidis

**Affiliations:** 1 First Department of Surgery, General Hospital Papageorgiou, Aristotle University of Thessaloniki, 56429 Thessaloniki, Greece; voula.hatzikomnitsa@yahoo.gr (P.C.); menelaospap.md@gmail.com (M.P.); evdo-t@hotmail.com (E.T.); dgiak@auth.gr (D.G.); papadvas@auth.gr (V.P.); alexgiakoustidis@gmail.com (A.G.); 2 Laboratory of Histology-Embryology, Department of Medicine, Faculty of Health Sciences, Aristotle University of Thessaloniki, 54124 Thessaloniki, Greece; thpapami@auth.gr

**Keywords:** colorectal cancer, chemotherapy, cardiotoxicity, FOLFOX, 5-Fluorouracil, CAPOX, echocardiography, cardiac biomarkers, arrhythmia, takotsubo cardiomyopathy, cardio-oncology, systematic review, heart failure, myocardial strain, multidisciplinary care

## Abstract

**Background/Introduction**: Colorectal carcinoma (CRC) belongs to the most commonly diagnosed malignancies to this date, ranking as third across the globe. In addition, CRC remains a leading cause of cancer-related deaths as it is ranked as the second most common cause of mortality. Therapeutic strategies for the management and treatment of CRC have made significant progress in the last two decades, with both adjuvant and neoadjuvant approaches playing critical roles in enhancing favorable outcomes with regimens like FOLFOX, CAPOX, and 5-FU-based therapies demonstrating effectiveness. Nevertheless, growing evidence indicates that these therapies may pose a risk of cardiotoxicity development. A systematic review will be conducted to map the mechanistic pathways of chemotherapy-induced in CRC in order to bridge oncology and cardiology perspectives, highlighting emerging diagnostic tools and long-term surveillance gaps. **Purpose**: The objective of this study is the investigation of the prevalence and characteristics of cardiovascular problems linked to frequently employed chemotherapy regimens, as well as to evaluate existing diagnostic and therapeutic approaches. **Methodology**: A thorough search across databases, including PubMed (MEDLINE), Embase, and Cochrane Library, was performed to locate articles published up to 2025. The final studies included in the review underwent quality assessment. **Results**: Fourteen qualifying studies, comprising both prospective trials and case reports from diverse geographies, were included. Cardiovascular outcomes including myocardial strain, arrhythmias, angina, heart failure, and Takotsubo cardiomyopathy were evaluated. The diagnostic methods assessed comprised echocardiography, cardiac biomarkers, and electrocardiograms. In the reviewed trials, chemotherapy-induced cardiotoxicity varied from asymptomatic ventricular strain to serious cardiac complications. The FOLFOX and 5-FU regimens were predominantly linked to adverse cardiac outcomes. Prompt identification by echocardiographic strain imaging and biomarker monitoring facilitated timely intervention. Case studies revealed that, given proper cardiological support, certain patients could safely recommence chemotherapy following recovery. No standardized cardiac screening protocol was identified among the trials. **Conclusions**: Chemotherapy for colorectal cancer may present considerable cardiovascular hazards, highlighting the necessity for routine cardiac monitoring prior to and throughout treatment. This systematic review promotes collaborative cardio-oncology strategies to reduce risk and enhance therapeutic safety.

## Simple Summary

The present systematic review explored how chemotherapy used to treat primary colorectal cancer, especially regimens like FOLFOX, CAPOX, and 5-FU/LV, can cause heart-related side effects. It includes 14 studies from various nations and unveiled that some individuals encountered minor concerns such as changes in heart strain, while others experienced more serious problems such as chest pain, arrhythmias, heart failure, or Takotsubo cardiomyopathy. Echocardiography and biomarkers were useful in detecting these alterations early. Case studies revealed that with careful monitoring and treatment, some patients were able to safely continue chemotherapy after recovering from cardiac toxicity. Overall, the study emphasizes the importance of regular cardiovascular exams before and during cancer therapy to improve patient safety and outcomes.

## 1. Introduction

Colorectal carcinoma (CRC) belongs to the most commonly diagnosed malignancies to this date, ranking as third across the globe [1]. In addition, CRC remains a leading cause of cancer-related deaths as it is ranked as the second most common cause of mortality [1,2]. Epidemiological studies project that by 2035, cases of colon and rectal cancer will increase by 71.5% and 60%, respectively [3]. The incline is mainly attributed to increase in aging of the general population, lifestyle factors, and disparities in access to screening and preventive care.

The majority of CRC cases are sporadic, arising from the accumulation of genetic and environmental factors over time. Around one-third of CRC patients exhibit familial clustering; however, only 5–16% of cases are associated with a germline pathogenic or likely pathogenic variation in a colorectal cancer predisposition gene. Hereditary colorectal cancer (HCRC) encompasses a group of illnesses categorized into two main categories, each exhibiting distinct clinical features: hereditary non-polyposis colorectal cancer (HNPCC) and hereditary polyposis colorectal cancer (HPCC) [1,4,5]. Lynch syndrome and familial adenomatous polyposis (FAP) are defined by germline mutations in DNA mismatch repair genes or changes in the APC gene [6]. Recent advancements in molecular profiling have shown the biological heterogeneity of colorectal cancer (CRC), resulting in its categorization into four consensus molecular subtypes (CMS): CMS1 (MSI-Immune), CMS2 (Canonical), CMS3 (Metabolic), and CMS4 (Mesenchymal). The genomic, epigenetic, and transcriptomic markers of these subtypes are distinct, influencing prognosis, therapeutic response, and tumour behaviour [7].

CRC screening is assessed by the use of fecal occult blood tests or endoscopic procedures, such as sigmoidoscopy or colonoscopy. Diagnosis is achieved by a combination of colonoscopy, biopsy, and imaging studies. Additional diagnostic approaches include biomarkers such carcinoembryonic antigen (CEA) and carbohydrate antigen but, their application is limited to monitoring disease progression rather than its development [8,9]. Multiple screening options are available, but high-quality evidence to indicate the best strategies is limited at the current stage [8].

Therapeutic strategies for the management and treatment of CRC have made significant progress in the last two decades, with both adjuvant and neoadjuvant approaches playing critical roles in enhancing favorable outcomes. Adjuvant therapy, which is typically administered in post-surgical resection, is designed for the elimination of any residual microscopic disease and decrease recurrence, rates with a particular emphasis on stage III and high-risk stage II CRC. This goal is reached by chemotherapy regimens including as FOLFOX (Folinic acid (also known as leucovorin), Fluorouracil (5-FU), and Oxaliplatin) or XELOX (CAPOX) (capecitabine and oxaliplatin combined). Moertel et al. proposed for the first time in the literature that mortality rates of stage III lymph node-positive colon cancer patients decreased by 33% after the administration of a 12-month regimen of 5-fluorouracil (5-FU) and levamisole [10]. In both stage III and high-risk stage II CRC, adjuvant chemotherapy with FOLFOX (fluorouracil, leucovorin, and oxaliplatin) regimen has been proved to reduce recurrence rates and enhance disease-free survival (DFS) [11]. In stage III patients, FOLFOX substantially increases DFS in comparison to 5-FU/LV alone, and it also exhibits a trend toward improved overall survival (OS). In high-risk stage II patients, FOLFOX, particularly when combined with oxaliplatin, exhibits an improvement in DFS and a reduction in recurrence risk [11].

On the other hand, neoadjuvant therapy, is particularly advantageous in the treatment of locally advanced rectal carcinoma. Chemoradiotherapy or total neoadjuvant therapy can enhance local management, improve tumour resectability, and occasionally support organ-preserving strategies. New evidence suggests that immunotherapy has great potential in the neoadjuvant setting for tumours with elevated microsatellite instability or mismatch repair deficiency [12,13]. A meta-analysis that was conducted by Gosavi R et al. (2021) suggested that neoadjuvant chemotherapy is a safe treatment option for the management of locally advanced colon cancer (LACC), indicating that tumour downstaging and increase in the rate of R0 resection have an oncological benefit. Consequently, neoadjuvant chemotherapy can be recommended as an alternative treatment option prior to surgery for clinically staged advanced colon malignancies (T4b), particularly when a clear resection margin is uncertain [14]. Even, with the therapeutic advancements present from the treatment options available, several factors must be taken into consideration of which adverse effects are included into the spectrum. Most notably, chemotherapy-induced cardiotoxicity is of great warning, which poses new challenges in the long-term management of CRC patients.

**Figure 1:**
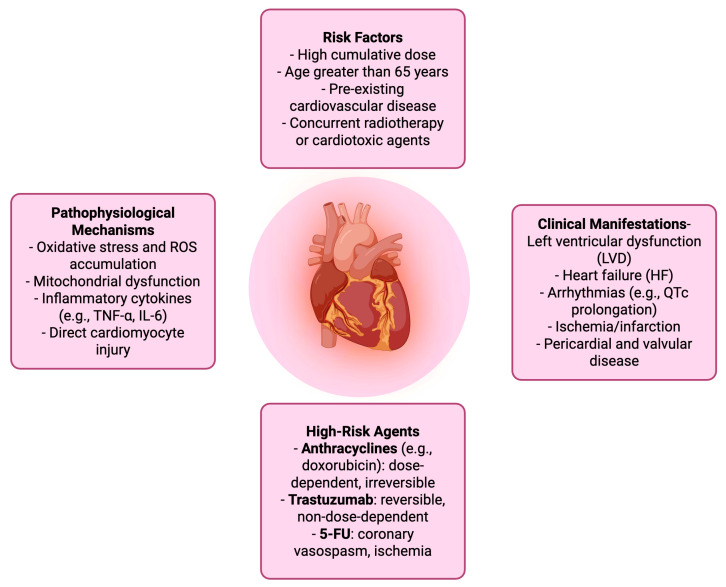
Chemotherapy-Related cardiotoxicity.

Chemotherapy-induced cardiotoxicity is a term referring to the spectrum of cardiac damage caused by cancer treatments and in particular from chemotherapeutic agents. It encircles both structural and functional cardiac impairments, ranging from asymptomatic changes in left ventricular ejection fraction (LVEF) to overt heart failure, arrhythmias, ischemia, and pericardial or valvular disease. A commonly used clinical definition includes a symptomatic drop in LVEF of ≥5% to below 55%, or an asymptomatic decline of ≥10% to below 55% [15]. The pathophysiology varies by drug class but often involves oxidative stress, mitochondrial dysfunction, and direct injury to cardiomyocytes. Anthracyclines like doxorubicin are known for dose-dependent, irreversible cardiotoxicity, while agents such as trastuzumab may cause reversible cardiac dysfunction [16,17,18,19,20]. Additionally, 5-FU is the second most common chemotherapeutic drug associated with cardiotoxicity after anthracyclines. It can manifest as chest pain, acute coronary syndrome/myocardial infarction or death [21]. The risk is influenced by cumulative dose, treatment duration, patient age, pre-existing cardiovascular conditions, and concurrent use of other cardiotoxic therapies. Early detection and monitoring are crucial, as cardiotoxicity can significantly impact both cancer prognosis and long-term cardiovascular health [15,22] (Table 1) (Figure 1).

Despite increasing recognition of chemotherapy-induced cardiotoxicity, its mechanistic underpinnings in the context of primary colorectal cancer remain poorly delineated. This systematic comprehensively synthesizes mechanistic evidence across diverse chemotherapy regimens specific to CRC, integrating molecular, imaging, and biomarker-based insights. By bridging oncology and cardiology perspectives, this work identifies critical gaps in early detection, long-term surveillance, and risk stratification laying the groundwork for future cardio-oncology integration and precision medicine approaches.

### Objective

The primary objective of the current systematic review is to investigate and summarize the current evidence present concerning the mechanisms and prevalence of cardiotoxicity arising from chemotherapy regimens that are used to treat primary CRC. This review endeavors to identify at-risk populations, evaluate early detection and prevention strategies, and delineate pharmacologic and pathophysiologic pathways that contribute to cardiac dysfunction in CRC patients undergoing chemotherapy. The ultimate objective is to enhance clinical outcomes.

## 2. Materials and Methods

In order to guarantee methodological transparency and rigour, this systematic review was implemented in accordance with the PRISMA (Preferred Reporting Items for Systematic reviews and Meta-Analyses) guidelines, as detailed in Supplementary Table S1. The review was not registered. To clarify the research query of this systematic review, a PICO (Population, Intervention, Comparator, Outcome) framework was employed, as detailed in Table 2.

### 2.1. Search Strategy

A strategic literature search was performed across multiple scientific databases, including PubMed (MEDLINE), Embase, and Cochrane Library, for the identification of the relevant studies published to this date. Keywords and MeSH terms related to CRC, chemotherapy regimens and cardiotoxicity were used for the creation of a Boolean string found in the Supplementary data Table S2. References were reviewed for identification of any additional relevant articles.

### 2.2. Study Selection—Eligibility Criteria

Inclusion and exclusion criteria were applied to ensure that only the most relevant and targeted articles were included during the screening process.

### 2.3. Inclusion Criteria

- Studies focusing on patients aged older than 19 years old diagnosed with primary CRC undergoing chemotherapy.
- Eligible interventions comprise commonly used chemotherapeutic agents such as fluoropyrimidines (5-fluorouracil and capecitabine), oxaliplatin, irinotecan, and regimens like FOLFOX, CAPEOX, FOLFIRI, and FOLFOXIRI.
- Studies must report cardiotoxicity outcomes including cardiac dysfunction, arrhythmias, myocarditis, QT prolongation, heart failure, or ischemic events in patients without a prior history of cardiovascular disease.
- Only clinical trials, cohort studies, case reports, cased series, case–control designs, and mechanistic studies published up to 2025 in English, with full-text availability, are considered for evaluation.

### 2.4. Exclusion Criteria

- Studies involving non-adult patients (under the age of 19 years old).
- Patients with metastatic CRC, other gastrointestinal malignancies, disease recurrence or non-cancer diagnoses.
- Interventions limited to radiotherapy or immunotherapy alone, or chemotherapy unrelated to CRC.
- Studies not reporting cardiovascular outcomes, or those involving patients with known cardiovascular disease.
- Additional filters must remove non-English studies without translation.
- Editorials, commentaries, abstracts, literature reviews and non-human or in vitro studies.

### 2.5. Screening Process

For the screening process a double-blind implementation was used for the elimination of decision influence between the two reviewers (S.T. and I.K.). The initial screening involved a systematic review of titles and abstracts to determine eligibility based on predefined inclusion and exclusion criteria. Any studies that referred to chemotherapy regimens for primary CRC and reported cardiotoxicity outcomes were shortlisted for the second round of screening. This stage excluded any publication that clearly involved pediatric patients, metastatic disease, radiotherapy or immunotherapy alone, or cardiovascular comorbidities.

**Figure 2:**
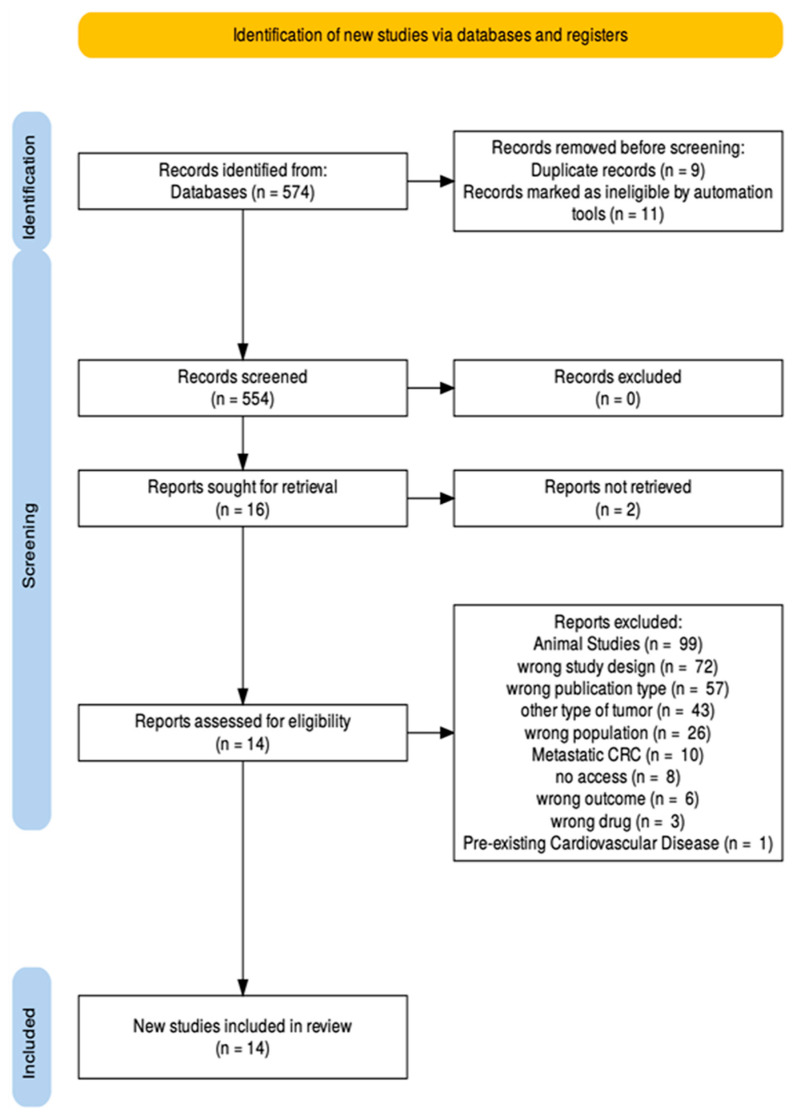
PRISMA flow diagram.

Moving on, full-text articles were retrieved for all potentially relevant studies. Each article underwent a second round of evaluation to confirm compliance with study design, publication date, language, and outcome specifications. Discrepancies in selection were resolved by consultation with a third investigator (M.P.) when necessary. The PRISMA flow diagram in Figure 2 outlines clearly the screening process taken place. From the final studies the relevant data were extracted and summarized into tables (Table 3, Table 4 and Table 5).

### 2.6. Quality Assessment

For the evaluation of the methodological strength and clinical relevance of the included studies, two validated appraisal tools were utilized: the Newcastle–Ottawa Scale (NOS) for cohort and observational studies, and the JBI Checklist for Case Reports and Case Series for descriptive accounts.

## 3. Results

### 3.1. Study Design and Geographical Scope of Included Research

The current study integrated data from a total of 14 studies conducted in nine different countries, including China, Italy, Poland, Hong Kong, Taiwan, Denmark, Greece, Canada and the United States, with the total sample size reaching 61,614 participants. The included studies compromised of a wide range of methodological types, including six prospective observational cohorts [23,24,25,26,27,28] to provide insights into cardiovascular outcomes among patients undergoing CRC chemotherapy. Four studies were retrospective cohort analyses [29,30,31,32], while Lee et al. (2022) [33] presented a population-based cohort analysis involving CRC patients with matched controls. Płońska-Gościniak et al. (2017) [25] was the only multicentre prospective cohort. Additional contributions include a single case series by McAndrew et al. (2021) [34] and two thorough case reports [35,36] which improve overall understanding of individual patient trajectories and unusual cardiovascular problems associated with treatment.

### 3.2. Quality Assessment—Newcastle-Ottawa Scale (NOS)

Three out of twelve cohort studies included achieved full scores using the Newcastle-Ottawa Scale (NOS), i.e., 9/9: Wong et al. (2025), Lee et al. (2022), and Huang et al. (2022) [29,32,33]. The high score of these studies can be largely attributed to their population-based designs made, matched control groups, long-term outcome tracking, and advanced statistical adjustments. Moving on, five studies that were assessed scored 7/9, including Wang (2021), Sonaglioni (2020), Gościniak (2017), Visvikis (2020), and Dyhl-Polk (2021), [23,24,25,26,27]. The reason for not reaching full score is due to lack of control cohorts being included but the studies overall retained strong internal validity and longitudinal outcome measurement. When it comes to Liu (2024) and Wang (2023) [30,31] they scored 8/9, that is c supported by retrospective designs and multivariate modeling for cardiotoxicity risk prediction. Cardiovascular outcomes reported showed increased heterogeneity, with some detecting subclinical cardiac dysfunction via speckle-tracking echocardiography and tissue Doppler, while others reported increased vascular burden post-chemotherapy [27] or elevated biomarkers signaling silent ischemia [26]. Large-scale cohort studies [29,32,33] found modest but statistically significant increases in cardiac events, particularly in older patients with comorbidities, and helped establish independent predictors of fluoropyrimidine-induced cardiotoxicity. A detailed overview of the NOS assessment criteria and scoring methodology is shown in Supplementary data Tables S3–S5.

### 3.3. Quality Assessment—JBI Checklist

All four case reports [Ben-Yakov (2017) [28], McAndrew (2021) [34], Vargo (2015) [36], and Sami (2025) [35]]; attained a perfect score (14/14) according to the critical evaluation criteria derived from the JBI checklist. Each case distinctly reported patient demographics, delineated a historical clinical history, and used precise diagnostic techniques such as echocardiography, angiography and cardiac MRI, when applicable. Interventions were accurately outlined and customized to address the specific cardiotoxic pathophysiological mechanisms, including vasospasm management, heart failure treatment, and inpatient chemotherapy rechallenge regimens. Outcomes pre and post chemotherapy were well presented, suggesting symptom relief and cardiac recovery in every instance. Cardiac adverse events, such as coronary vasospasm, cardiogenic shock, and Takotsubo cardiomyopathy, were examined unambiguously, with each case providing significant clinical insights that enhance best clinical practice for 5-FU rechallenge and cardio-oncology monitoring. These articles collectively provide methodologically robust and meaningful clinical observations that strengthen the evidence base for the management of fluoropyrimidine-induced cardiotoxicity. A detailed overview of the JBI checklist can be accessed in Supplementary data Table S6.

## 4. Discussion

**Figure 3:**
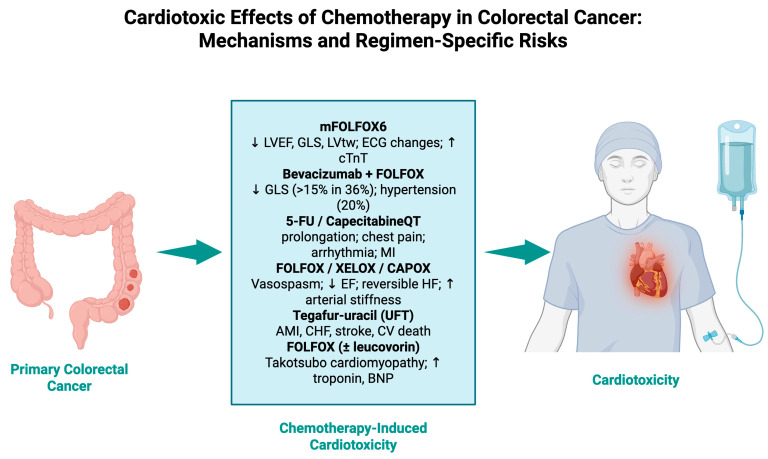
Cardiotoxic effects of chemotherapy in colorectal cancer: mechanisms and regimen-specific risks.

The intersection of oncology and cardiology is gaining traction, especially in light of new data that chemotherapy-induced cardiotoxicity may be more common and complicated than previously thought. In this analysis, we analyzed data from a wide range of international studies to demonstrate the complexities and diversity of cardiovascular outcomes related to CRC treatment (Figure 3).

### 4.1. Chemotherapy Regimens and Treatment Combinations

The examined chemotherapy regimens primarily involved fluoropyrimidine-based treatments, namely 5-FU, capecitabine, and tegafur-uracil (UFT) either alone or in combination with agents such as oxaliplatin, leucovorin, cisplatin, or bevacizumab. Notable combinations included FOLFOX [23,24,27], CAPOX [34], XELOX [27] and mFOLFOX6 [23]. The diversity in regimens allowed for comparisons of cardiotoxic risk across both monotherapies and multidrug protocols, as evidenced by analyses [25,29,31,33,37]. Tegafur-uracil (UFT) was specifically examined in Huang et al. (2022) [32], allowing for a unique comparison to non-UFT protocols. Furthermore, pharmacological drugs such as leucovorin and bevacizumab, particularly in combination forms such as FOLFOX plus Bevacizumab [24,31] and cisplatin [26] were included, which contributed to risk stratification across regimens. Several case reports also identified specific adverse cardiac events associated with 5-FU-based therapy [28,35,36].

### 4.2. Advanced Imaging Techniques for Detecting Chemotherapy-Induced Cardiotoxicity

Cardiotoxicity outcomes ranged from minor subclinical alterations to major cardiac events. Several studies have shown that sophisticated imaging techniques such as 3D and 2D speckle-tracking echocardiography (STE) can detect early cardiac damage. Wang et al. (2021) [23] and Sonaglioni et al. (2020) [24] found reductions in global longitudinal strain (GLS) and left ventricular twist (LVtw) despite preserved left ventricular ejection fraction (LVEF), implying that strain-based imaging may be more sensitive for detecting early cardiotoxicity. Płońska-Gościniak et al. (2017) [25] utilized tissue Doppler echocardiography to assess transitory QT interval prolongation and small changes in cardiac velocities 12 months post-chemotherapy.

### 4.3. Clinical Manifestations and Cardiotoxic Sequelae

Chemotherapy-induced cardiotoxicity in CRC patients presents a wide clinical spectrum, ranging from transient, asymptomatic cardiovascular changes to severe and potentially life-threatening cardiac events. Commonly addressed clinical presentations include chest pain, hypertension, arrhythmias, and myocardial ischemia. Dyhl-Polk et al. (2021) [26] reported silent ischemia in 14.1% of CRC patients receiving their first 5-FU infusion. Notably, elevations in copeptin levels were more reliable than troponin as markers of ischemic stress, suggesting copeptin’s role as a promising biomarker for early cardiovascular adverse events.

Acute, but rare, manifestations, underscored mainly in case studies, include reversible cardiomyopathy, cardiogenic shock, and stress-induced cardiomyopathy, which are typically triggered by capecitabine- or 5-FU-based regimens. McAndrew et al., 2021 [34], and Sami et al., 2025 [35], reported complete recovery of cardiac function in affected patients, followed by successful chemotherapy rechallenge under monitored conditions. Such evidence highlights the feasibility of continuing chemotherapy safely with appropriate cardiovascular surveillance and interdisciplinary management.

Population-based research in Hong Kong, Taiwan, and China found strong evidence of long-term vascular risks. Wong et al. (2025) [29] found a 1.06% incidence of major adverse cardiovascular events (MACE), with no increased risk compared to matched controls or between 5-FU and capecitabine. Lee et al. (2022) [33] and Huang et al. (2022) [32] found significantly higher rates of stroke, heart failure, and ischemic heart disease in older patients and those treated with UFT, particularly in stage III CRC. These risks were heightened by prevalent cardiovascular comorbidities such as diabetes, hypertension, and dyslipidemia.

Biomarkers and risk-stratification methods proved useful in predicting and monitoring cardiotoxicity. Increased cardiac troponins and copeptin levels have been correlated with ischemic events, whereas Liu et al. (2024) [30] reported that the systemic immune-inflammation index (SII) was a good predictor of cardiotoxicity in patients with low monocyte cell count. Wang et al. (2023) [31] developed a risk nomogram that includes characteristics like age ≥ 60, BMI ≥ 22.97 kg/m^2^, ≤3 chemotherapy cycles, and concurrent bevacizumab usage, which are all independently related to elevated cardiotoxicity risk.

These findings line up with a wider revolution in oncological care that prioritizes increased survival rates and quality of life, and the reduction in long-term toxicities. As treatment methods become increasingly precise and survival rates rise, understanding the concealed costs of therapy, particularly those impacting cardiac health, has become essential. Our findings highlight a crucial necessity to reconsider current monitoring systems and risk assessment methodologies.

### 4.4. Nanotechnology-Driven Innovations in Cardiotoxicity Prevention and Monitoring

In addition, nanotechnology is set to play a transformative role in the early identification, prevention, and mitigation of chemotherapy-related cardiotoxicity. Nanoparticle-mediated drug delivery systems possess the capability to specifically target tumour cells while preserving cardiac tissue, therefore minimizing off-target harm [38,39,40]. Engineered nanocarriers can be formulated to co-deliver chemotherapeutic drugs with cardioprotective substances, enhancing therapeutic efficacy and safety. Therapeutic drugs can be given directly to tumour areas while preserving healthy tissues, reducing off-target effects, by functionalizing nanoparticles (NPs) with ligands or antibodies that attach to receptors on cancer cells [41]. Moreover, nanosensors and nanobiosensors could provide real-time monitoring of cardiac biomarkers at the cellular level, hence enabling early identification of subclinical myocardial injury. Future investigations should examine the incorporation of nanotechnology into cardio-oncology treatments, focusing on biocompatibility, long-term safety, and translational viability [41,42,43,44].

### 4.5. Limitations

This systematic review is restricted by several inherent limitations that necessitate caution in interpretation. The diversity in study designs from observational cohorts to descriptive case reports introduces heterogeneity in diagnostic protocols, cardiotoxicity definitions and outcome reporting. Lack of randomized controlled trials (RCTs) weakens causal inference, while limited access to baseline cardiovascular data in some cohorts may lead to underestimation of potential subclinical toxicities. Geographic clustering in East Asia and North America decreases the pertinence of findings to global populations, particularly in underserved regions. Moreover, shorter follow-up durations hinder comprehensive understanding of long-term cardiovascular risk, and the publication bias intrinsic in case reports may skew observed outcomes towards more dramatic or successful events.

### 4.6. Prospective Research Directions

Future research should prioritize the implementation of extensive, multicenter RCTs to conclusively associate chemotherapy methods with cardiotoxic effects in colorectal cancer patients. Implementing systematic pre-treatment cardiac evaluations, including baseline echocardiogram, full biomarker panels, and genetic screening, would improve the precision of patient risk classification. Advanced diagnostic modalities such as 3-D speckle-tracking echocardiography and novel serum biomarkers such as copeptin necessitate systematic validation as early indicators of myocardial injury. Moreover, prospective longitudinal cohorts drawn from diverse, representative populations with follow-up periods extending beyond five to ten years are essential to elucidate the chronic cardiotoxic risk and overall survival. The integration of oncologic and cardiologic care through multidisciplinary cardio-oncology programs, which combine treatment decision-making with continuous cardiac surveillance, has the potential to refine safety thresholds and inform evidence-based rechallenge strategies. Thus, looking forward, future aspects of research should also explore AI and machine learning (ML) algorithms as predictive models for the risk of cardiotoxicity development, integrating clinical, imaging, and genomic data. Also, pharmacogenomic profiling is a good addition for the identification of patient-specific susceptibilities and guide safer chemotherapy selection. The development of cardioprotective agents and targeted interventions for the mitigation of myocardial injury without compromising oncologic efficacy will provide beneficial results as well as the incorporation of real-time wearable cardiac monitoring technologies to detect early functional changes during chemotherapy administration. Together medical societies can create a global harmonization of cardio-oncology guidelines, by enabling standardized risk assessment and surveillance protocols across healthcare systems. These future directions will aim to transform cardiotoxicity management from reactive to proactive, ensuring safer, more personalized care for colorectal cancer patients undergoing chemotherapy.

## 5. Conclusions

The findings of this systematic review demonstrate that fluoropyrimidine-based chemotherapy regimens, including 5-fluorouracil, capecitabine, and tegafur-uracil, either alone or in combination with oxaliplatin (FOLFOX, CAPOX/XELOX), carry a measurable risk of cardiotoxicity in patients with primary CRC. Cardiac clinical manifestations span subclinical myocardial strain abnormalities diagnosed by speckle-tracking and tissue Doppler echocardiography to overt clinical adverse events, such as coronary vasospasm, arrhythmias, ischemia, reversible heart failure, and stress-induced cardiomyopathy. Serum biomarkers, such as hs-Troponin-I, N-terminal pro-B-type natriuretic peptide and copeptin, and advanced imaging modalities permit earlier identification and stratification of myocardial injury, while population-based cohorts and retrospective analyses underscore that older age, baseline cardiovascular comorbidities, and specific therapeutic combinations, such as concurrent bevacizumab or limited chemotherapy cycles, significantly elevate the risk. Case reports further establish that, under meticulous cardio-oncology surveillance, many patients can safely resume to potentially curative chemotherapy regimens after an acute cardiotoxic event.

To alleviate these risks and improve patient outcomes, integration of standard cardiac monitoring protocols, including baseline and serial echocardiographic strain imaging, targeted serum biomarker surveillance, and individualized risk-stratification nomograms, should become fundamental to CRC care. Prospective, multicenter studies with extended follow-up periods are required to further validate early detection diagnostic methods, define long-term cardiovascular sequelae, and evaluate cardioprotective interventions. Establishing multidisciplinary cardio-oncology pathways will be crucial to balance chemotherapeutic efficacy with cardiovascular safety, ultimately enhancing both survival rates and quality of life for patients undergoing treatment for primary colorectal malignancies.

## Figures and Tables

**Table 1:** Overview of chemotherapy-related cardiotoxicity.

Domain	Details
Definition	Cardiac dysfunction resulting from cancer therapy, particularly chemotherapeutic agents. Includes structural and functional damage such as LVEF decline and HF.
Diagnostic Criteria	- Symptomatic: ≥5% drop in LVEF to <55% - Asymptomatic: ≥10% drop in LVEF to <55%
Clinical Manifestations	- Left ventricular dysfunction (LVD) - Heart failure (HF) - Arrhythmias (e.g., QTc prolongation) - Ischemia/infarction - Pericardial and valvular disease
Pathophysiological Mechanisms	- Oxidative stress and ROS accumulation - Mitochondrial dysfunction - Inflammatory cytokines (e.g., TNF-α, IL-6) - Direct cardiomyocyte injury
High-Risk Agents	- Anthracyclines (e.g., doxorubicin): dose-dependent, irreversible - Trastuzumab: reversible, non-dose-dependent - 5-FU: coronary vasospasm, ischemia
Risk Factors	- High cumulative dose - Age >65 years - Pre-existing cardiovascular disease - Concurrent radiotherapy or cardiotoxic agents
Monitoring Modalities	- Echocardiography (LVEF, GLS) - Cardiac biomarkers (troponin, BNP) - ECG - Cardiac MRI
Preventive Strategies	- Dose limitation and scheduling adjustments - Use of cardioprotective agents (e.g., dexrazoxane) - Baseline and serial cardiac assessment
Management Approaches	- Temporary or permanent discontinuation of culprit agent - Initiation of HF therapy (e.g., ACE inhibitors, beta-blockers) - Multidisciplinary cardio-oncology care

**Table 2:** PICO table.

Component	Description
P (Population)	Adult patients diagnosed with primary colorectal cancer undergoing chemotherapy treatment
I (Intervention)	Administration of chemotherapy regimens (FOLFOX, XELOX, 5-FU, capecitabine, oxaliplatin)
C (Comparison)	CRC patients not receiving chemotherapy, receiving non-cardiotoxic agents, or baseline cardiac function pre-chemotherapy
O (Outcome)	Incidence, mechanisms, and severity of cardiotoxicity (myocardial injury, arrhythmias, heart failure), as well as detection and monitoring strategies

**Table 3:** Summary of included studies evaluating chemotherapy-induced cardiotoxicity in primary colorectal cancer patients. Abbreviations: Increase, ↑; Decrease, ↓.

Authors (Year)	Country	Study Design	Sample Size	Population Characteristics	Chemotherapy Regimen	Cardiotoxicity Outcome(s)
Wang et al. (2021) [23]	China	Prospective observational	30	Adults aged 37–64, diagnosed with primary CRC, no preexisting CVD	mFOLFOX6	Reduced LVEF, GLS, LVtw; ECG changes; ↑ cTnT levels
Sonaglioni et al. (2020) [24]	Italy	Prospective observational	25	Adults (mean age 71.8 ± 7.5), mCRC, normotensive	Bevacizumab + FOLFOX	>15% decrease in GLS (36%); new-onset hypertension (20%)
Płońska-Gościniak et al. (2017) [25]	Poland	Prospective multicentre	25	Adults (12F), mean age 61.3 y, CRC (adenocarcinoma)	5-FU or capecitabine regimens	↓ S’IVS, S’lat, E’sept; transient QT prolongation
Dyhl-Polk et al. (2021) [26]	Denmark	Prospective observational	108	CRC and anal cancer patients receiving 5-FU for the first time	5-FU ± cisplatin, FOLFOX, FOLFIRI	ST changes, ischemia, arrhythmias, ↑ copeptin, rare ↑ troponin
Visvikis et al. (2020) [27]	Greece	Prospective observational	70	Non-metastatic CRC (Stage II & III); no prior cardiac disease	FOLFOX or XELOX (Oxaliplatin + 5-FU/Capecitabine)	↑ arterial stiffness: PWV, Aix75, SBP; no change in EF
Ben-Yakov et al. (2017) [28]	Canada/USA	Case report	1	54-year-old male with rectal cancer, HTN, hyperlipidemia, smoker	5-FU infusion	Chest pain, hyperacute T waves, reversible ST changes, vasospasm
Wong et al. (2025) [29]	Hong Kong	Retrospective cohort w/matching	21, 216 (After propensity score matching)	Adults with CRC, balanced for CVD risk factors	5-FU and capecitabine	1.06% experienced major adverse cardiovascular event (MACE); no ↑ CV risk vs. controls; no difference between drugs
Liu et al. (2024) [30]	China	Retrospective cohort	754	CRC pts on 5-FU-based regimens, normal cardiac baseline, stratified by monocytes	5-FU-based regimens	Chest pain, arrhythmias, ECG/ST-T changes, ↑ cardiac enzymes
Wang et al. (2023) [31]	China	Retrospective cohort	916	CRC patients, 5-FU or capecitabine-based chemo, no severe CVD at baseline	5-FU or capecitabine	Chest pain, arrhythmia, dyspnea, ST-T changes, myocardial infarction
Huang et al. (2022) [32]	Taiwan	Nationwide retrospective cohort	32, 35	Stage II–III CRC post-resection, treated with UFT, non-UFT, or mixed 5-FU regimens	Tegafur-uracil (UFT), non-UFT, mixed	AMI, LTA, CHF, ischemic stroke, CV death
Lee et al. (2022) [33]	Hong Kong	Population-based cohort	1037 CRC + 5078 controls	Stage II–III CRC survivors post-radical surgery, receiving adjuvant fluoropyrimidine-based chemo	Fluoropyrimidines (5-FU/capecitabine ± oxaliplatin)	Ischemic heart disease, heart failure, cardiomyopathy, stroke
McAndrew et al. (2021) [34]	Canada	Case series	2	CRC patients on CAPOX with no prior cardiac history; acute HF within days of first cycle	CAPOX (Capecitabine + Oxaliplatin)	Cardiogenic shock, reversible heart failure, ↓ LVEF < 20%, elevated lactate
Sami et al. (2025) [35]	USA	Case report	1	74-year-old woman with sigmoid adenocarcinoma and severe anxiety	FOLFOX (5-FU + leucovorin + oxaliplatin)	Takotsubo cardiomyopathy (TCM); LV dysfunction; elevated troponin and BNP
Vargo et al. (2015) [36]	USA	Case report	1	48-year-old woman with stage IIIc colon cancer, no cardiac risk factors	FOLFOX (5-FU + oxaliplatin)	Vasospastic angina during 5-FU infusion; recurrent chest pain

**Table 4:** Overview of included studies investigating chemotherapy-associated cardiotoxicity in primary colorectal cancer patients. Abbreviations: Increase, ↑; Decrease, ↓.

Authors (Year)	Measurement Tools	Timing of Outcome Assessment	Key Findings
Wang et al. (2021) [23]	3D speckle-tracking echocardiography, serum cTnT	Before chemo; after cycles 1, 6, and 12	MCI showed highest sensitivity for early cardiotoxicity; significant decline in strain measures & ↑ cTnT
Sonaglioni et al. (2020) [24]	2D STE, BP, ECG, HS-cTnI	Baseline, 3 months, 6 months	GLS impaired over 6 months; no LVEF changes; 2D-STE effective for early cardiotoxicity detection
Płońska-Gościniak et al. (2017) [25]	Tissue Doppler Echo, ECG	Baseline, 3, 6, and 12 months	Chemotherapy utilizing 5-FU or capecitabine in CRC patients did not influence the conduction system, left ventricular structural features, or systolic function as assessed by left ventricular ejection fraction, nor is it linked to cardiovascular events in the following 12 months. Chemotherapy with 5-FU or capecitabine in CRC patients may induce minor alterations in cardiac function, detected exclusively through tissue analysis. Doppler echocardiography conducted after a duration of 12 months. Transient QT prolongation occurs during CTX and resolves upon discontinuation of CTX.
Dyhl-Polk et al. (2021) [26]	Holter ECG, 12-lead ECG, copeptin, troponin I	Before chemo and during 1st & 3rd/4th cycles	14.1% had silent ischemia during first 5-FU infusion; 5.6% developed ACS; copeptin ↑ during treatment, troponin rarely elevated
Visvikis et al. (2020) [27]	ECG, Echo, SphygmoCor tonometry, Complior PWV, BP monitoring	Before and after full chemo regimen (6–12 cycles)	Significant post-chemo ↑ in Aix75, PWVc-f, PWVc-r (*p* < 0.001); changes independent of regimen; ↑ vascular burden post-adjuvant chemo requires follow-up
Ben-Yakov et al. (2017) [28]	ECG, coronary angiography, troponin, LV angiogram	During 2nd cycle of 5-FU infusion	Coronary vasospasm mimicking STEMI; no obstructive CAD; LV dilation with global hypokinesis; resolved with NTG and CCB; first EM case report of 5-FU vasospasm
Wong et al. (2025) [29]	Clinical records, troponin, survival analysis	1-year follow-up	Fluoropyrimidines did not ↑ MACE risk; no significant difference between 5-FU and capecitabine
Liu et al. (2024) [30]	Clinical symptoms, ECG, echo, serum markers, SII	During treatment and up to 4 weeks post-completion	SII positively correlated with cardiotoxicity in low-monocyte subgroup; threshold effect observed in mid-tertile
Wang et al. (2023) [31]	Clinical records, ECG, echo, labs, CTCAE grading	During chemo and up to 4 weeks post-treatment	Age > 60, BMI ≥ 22.97, ≤3 cycles of chemo, and use of bevacizumab ↑ risk of 5-FU–induced cardiotoxicity; nomogram developed
Huang et al. (2022) [32]	TCR, NHIRD, TDR linkage; SIPTW adjusted modeling	Up to 14 years post-diagnosis	UFT group had ↑ heart failure, stroke, AMI risk vs. other groups; strongest in stage III and age ≥ 70; caution advised for this subset
Lee et al. (2022) [33]	Electronic medical records, ICD codes, competing risk modeling	1–10 years post-treatment	Adjusted HR for CVD in chemo-treated CRC vs. controls = 2.11; strongest risk factors: age, male sex, diabetes, HTN, dyslipidemia; stroke risk notably elevated
McAndrew et al. (2021) [34]	ECG, TTE, Cardiac MRI, CT, coronary angiography, lab biomarkers	Day 3–7 post treatment initiation	Severe reversible cardiomyopathy; full cardiac recovery without infarction, vasospasm or myocarditis; Capecitabine implicated in acute HF without ST changes
Sami et al. (2025) [35]	ECG, TTE, coronary angiography, ventriculography, BNP, troponin	3 days post-cycle 1; 1 month post-rechallenge	TCM resolved with conservative therapy; successful rechallenge with telemetry monitoring and no recurrence; highlights need for cardiac surveillance
Vargo et al. (2015) [36]	ECG, echo, cardiac catheterization, troponin	During all 10 cycles of 5-FU infusion	Rechallenge successful with inpatient cardiac monitoring, nitrates, and calcium channel blockers; no troponin leak or ST changes; no delayed toxicity at 10-month follow-up

**Table 5:** Quantitative summary of reported cardiotoxicity risks stratified by chemotherapy regimen in primary colorectal cancer patients. ^1^ Regimen abbreviations are defined in the Abbreviations list. ^2^ Total sample size is the sum of participants across studies reporting the regimen. ^3^ Incidence/risk values are as reported in the original studies; ranges reflect variability in definitions and endpoints. ^4^ References correspond to those in the main reference list. Abbreviations: GLS, global longitudinal strain; LVtw, left ventricular twist; LVEF, left ventricular ejection fraction; HF, heart failure; AMI, acute myocardial infarction; ACS, acute coronary syndrome; MACE, major adverse cardiovascular events, ↑, Increase; ↓, Decrease.

Chemotherapy Regimen ^1^	Studies Reporting, *n*	Total Sample Size ^2^	Reported Cardiotoxicity Incidence/Risk ^3^	Commonly Reported Cardiac Events	Representative References ^4^
FOLFOX (5-FU + leucovorin + oxaliplatin)	6	~1200	5–36% (higher for subclinical strain changes)	↓ GLS, LVtw, vasospastic angina, arrhythmias, hypertension	[23,27,36]
CAPOX/XELOX (capecitabine + oxaliplatin)	2	72	2.8–100% in case series	Acute HF, cardiogenic shock, ↓ LVEF	[27,34]
5-FU monotherapy/infusional	5	>22,000	1.06% MACE in large cohorts; up to 14% silent ischemia	Chest pain, silent ischemia, vasospasm, arrhythmias	[26,28,29]
Capecitabine monotherapy	3	~1000	Similar to 5-FU in large cohorts; rare acute HF in case reports	Acute HF, arrhythmias, ischemia	[29,34]
Tegafur–uracil (UFT)	1	32,351	Increased risk of HF, stroke, AMI vs. non-UFT regimens (adjusted HRs significant in elderly, stage III)	HF, stroke, AMI	[32]
5-FU + bevacizumab	2	~150	20% new-onset hypertension; increased cardiotoxicity risk in predictive models	Hypertension, ischemia	[24,31]
5-FU + cisplatin	1	108	14.1% silent ischemia; 5.6% ACS	Silent ischemia, ACS	[26]

## Data Availability

The data presented in this study are available in this article and Supplementary Materials.

## Supplementary Materials

The following supporting information can be downloaded at: https://www.mdpi.com/article/10.3390/cancers17193129/s1, Table S1: PRISMA 2020 Checklist [45]; Table S2: Boolean String; Table S3: Criteria; Table S4: Quality Assesment Scale—Newcastle-Ottawa Scale (NOS); Table S5: Quality Assessment Scale—Newcastle-Ottawa Scale (NOS); Table S6: Quality Assesment Scale—JBI checklist.
