# Supplementary material for: Unveiling the Mechanisms for the Development of Cardiotoxicity Following Chemotherapy Regimens Administration for Primary Colorectal Cancer: A Systematic Review
Source: Cancers (Basel). 2025 Sep 26;17(19):3129. doi: 10.3390/cancers17193129 (PMC12523954; doi:10.3390/cancers17193129)
Supplement: Supplementary file 1 [file cancers-17-03129-s001.zip › Tables S2-S6. Data Crdiotoxicity CRC.pdf]

Table S2. Boolean String.

Search String for Systematic Review on Cardiotoxicity Induced by Chemotherapy in Colorectal Cancer
((("leucovorin"[Supplementary Concept] OR "leucovorin"[All Fields] OR "leucovorin calcium"[All Fields] OR "leucovorin"[MeSH Terms] OR ("leucovorin"[All Fields] AND "calcium"[All Fields])) AND ("leucovorin"[Supplementary Concept] OR "leucovorin"[All Fields] OR "folinic acid"[All Fields] OR "leucovorin"[MeSH Terms] OR ("folinic"[All Fields] AND "acid"[All Fields])) AND ("fluorouracil"[Supplementary Concept] OR "fluorouracil"[All Fields] OR "fluorouracil"[MeSH Terms] OR "fluorouracile"[All Fields] OR "fluorouracils"[All Fields]) AND ("oxaliplatin"[Supplementary Concept] OR "oxaliplatin"[All Fields] OR "oxaliplatin"[MeSH Terms] OR "oxaliplatine"[All Fields] OR "oxaliplatin s"[All Fields])) OR "FOLFOX"[All Fields] OR (("chemotherapy s"[All Fields] OR "drug therapy"[MeSH Terms] OR ("drug"[All Fields] AND "therapy"[All Fields]) OR "drug therapy"[All Fields] OR "chemotherapies"[All Fields] OR "drug therapy"[MeSH Subheading] OR "chemotherapy"[All Fields]) AND ("clinical protocols"[MeSH Terms] OR ("clinical"[All Fields] AND "protocols"[All Fields]) OR "clinical protocols"[All Fields] OR "regimen"[All Fields] OR "regimens"[All Fields] OR "regimen s"[All Fields])) OR "CAPEOX"[All Fields] OR ((("capecitabine"[Supplementary Concept] OR "capecitabine"[All Fields] OR "capecitabin"[All Fields] OR "capecitabine"[MeSH Terms]) AND ("oxaliplatin"[Supplementary Concept] OR "oxaliplatin"[All Fields] OR "oxaliplatin"[MeSH Terms] OR "oxaliplatine"[All Fields] OR "oxaliplatin s"[All Fields])) OR "FOLFIRI"[All Fields] OR (("leucovorin"[Supplementary Concept] OR "leucovorin"[All Fields] OR "leucovorin"[MeSH Terms] OR "leucovorine"[All Fields]) AND ("fluorouracil"[Supplementary Concept] OR "fluorouracil"[All Fields] OR "5 fu"[All Fields] OR "fluorouracil"[MeSH Terms]) AND ("irinotecan"[Supplementary Concept] OR "irinotecan"[All Fields] OR "irinotecan"[MeSH Terms] OR "irinotecan s"[All Fields])) OR "FOLFOXIRI"[All Fields] OR (((("leucovorin"[Supplementary Concept] OR "leucovorin"[All Fields] OR "leucovorin"[MeSH Terms] OR "leucovorine"[All Fields]) AND ("fluorouracil"[Supplementary Concept] OR "fluorouracil"[All Fields] OR "5 fu"[All Fields] OR "fluorouracil"[MeSH Terms]) AND ("oxaliplatin"[Supplementary Concept] OR "oxaliplatin"[All Fields] OR "oxaliplatin"[MeSH Terms] OR "oxaliplatine"[All Fields] OR "oxaliplatin s"[All Fields]) AND ("irinotecan"[Supplementary Concept] OR "irinotecan"[All Fields] OR "irinotecan"[MeSH Terms] OR "irinotecan s"[All Fields])) OR ("folfirinox"[Supplementary Concept] OR "folfirinox"[All Fields]) OR ("fluorouracil"[Supplementary Concept] OR "fluorouracil"[All Fields] OR "fluorouracil"[MeSH Terms] OR "fluorouracile"[All Fields] OR "fluorouracils"[All Fields])) AND ("leucovorin"[Supplementary Concept] OR "leucovorin"[All Fields] OR "leucovorin"[MeSH Terms] OR "leucovorine"[All Fields]) AND ("irinotecan"[Supplementary Concept] OR "irinotecan"[All Fields] OR "irinotecan"[MeSH Terms] OR "irinotecan s"[All Fields]) AND ("oxaliplatin"[Supplementary Concept] OR "oxaliplatin"[All Fields] OR "oxaliplatin"[MeSH Terms] OR "oxaliplatine"[All Fields] OR "oxaliplatin s"[All Fields])))) AND ("colorectal neoplasms"[MeSH Terms] OR ("colorectal"[All Fields] AND "neoplasms"[All Fields]) OR "colorectal neoplasms"[All Fields] OR ("colorectal"[All Fields] AND "cancer"[All Fields]) OR "colorectal cancer"[All Fields]) AND ("cardiotoxic"[All Fields] OR "cardiotoxicity"[MeSH Terms] OR "cardiotoxicity"[All Fields] OR "cardiotoxicities"[All Fields] OR "cardiotoxity"[All Fields])) AND ((y_10[Filter]) AND (excludepreprints[Filter] OR medline[Filter]) AND (casereports[Filter] OR clinicalstudy[Filter] OR clinicaltrial[Filter] OR clinicaltrialphasei[Filter] OR clinicaltrialphaseii[Filter] OR clinicaltrialphaseiii[Filter] OR clinicaltrialphaseiv[Filter] OR comparativestudy[Filter] OR controlledclinicaltrial[Filter] OR multicenterstudy[Filter] OR observationalstudy[Filter] OR randomizedcontrolledtrial[Filter]) AND (english[Filter]))

Table S3. Criteria.

Criteria	Inclusion	Exclusion
**Population**	Patients aged 19 years and older diagnosed with primary colorectal cancer (CRC) receiving chemotherapy	Studies on patients under 19, metastatic CRC, other gastrointestinal cancers, or non-cancer patients,recurrence
**Intervention**	Chemotherapy regimens used in primary CRC, including fluoropyrimidines (5-FU, capecitabine) oxaliplatin, irinotecan FOLFOX, CAPEOX, FOLFIRI, FOLFOXIRI)	Studies addressing radiotherapy or immunotherapy alone, or chemotherapy for unrelated cancers
**Outcome Measures**	Cardiotoxicity outcomes, such as cardiac dysfunction, arrhythmias, myocarditis, QT prolongation, heart failure, ischemic complications, patients with no history of cardiovascular disease (CVD)	Studies not reporting cardiovascular events or focusing solely on non-cardiac toxicity, Studies of patients with known CVD
**Study Design**	Randomized controlled trials (RCTs), cohort studies, case-control studies, case reports, mechanistic studies	Editorials, commentaries, conference abstracts, animal studies, or in vitro/laboratory-based studies, literature reviews
**Time Frame**	Studies up to 2025, ensuring relevance to modern chemotherapy protocols	-
**Language**	Studies available in English, with full-text access	Non-English studies without accessible translations

Table S4. Quality Assesment Scale—Newcastle-Ottawa Scale (NOS).

Study	1. Representativeness of Cohort	2. Selection of Non-Exposed Cohort	3. Ascertainment of Exposure	4. Outcome Absent at Start
Wang et al. (2021)	★	✖ (no control group)	★ (chemotherapy regimen confirmed)	★
Sonaglioni et al. (2020)	★	✖ (no control group)	★ (clinical + echocardiography)	★
Płońska-Gościniak et al. (2017)	★	✖ (no comparison/control group)	★ (confirmed CTX regimen)	★
Wong et al. (2025)	★	★	★	★
Liu et al. (2024)	★	✖ (no control group)	★ (confirmed 5-FU regimen)	★
Wang et al. (2023)	★	✖ (no non-exposed group)	★ (verified treatment records)	★
Lee et al. (2022)	★	★	★	★
Huang et al. (2022)	★	★	★	★
Dyhl-Polk et al. (2021)	★	✖ (no control group)	★ (verified 5-FU regimen + ECG)	★

**Visvikis et al. (2020)**	★	✕ (no control group)	★ (confirmed CTX regimen)	★

Table S5. Quality Assessment Scale—Newcastle-Ottawa Scale (NOS).

Study	5. Comparability of Cohorts	6. Outcome Assessment	7. Follow-Up Duration	8. Adequacy of Follow-Up	Total Stars
**Wang et al. (2021)**	★ (matched baseline stats)	★	★	★ (longitudinal sampling at 4 points)	**7 to 9**
**Sonaglioni et al. (2020)**	★ (basic demographics provided)	★	★ (6-month follow-up)	★ (full follow-up reported)	**7 to 9**
**Płońska-Gościniak et al. (2017)**	★ (stratified CTX subgroups)	★	★ (12-month follow-up)	★ (all pts completed follow-up)	**7 to 9**
**Wong et al. (2025)**	★★	★	★	★	**9 to 9**
**Liu et al. (2024)**	★★ (multivariate + interaction modeling)	★ (ECG, biomarkers, physician-confirmed)	★ (up to 4 wks post-chemo)	★ (complete cohort retained)	**8 to 9**
**Wang et al. (2023)**	★★ (LASSO + logistic modeling)	★	★ (assessed up to 4	★ (complete cohort analysis)	**8 to 9**

			weeks post-chemo)		
**Lee et al. (2022)**	★★	★	★ (≥10 years)	★ (low attrition, registry-based)	**9 to 9**
**Huang et al. (2022)**	★★	★	★	★	**9 to 9**
**Dyhl-Polk et al. (2021)**	★ (matched CV risk baseline)	★ (Holter + biomarkers)	★ (2 cycles + longitudinal)	★ (minimal attrition)	**7 to 9**
**Visvikis et al. (2020)**	★ (subgroup analysis by regimen)	★ (tonometry + echo + ECG)	★ (baseline & post chemo)	★ (all completed follow-up)	**7 to 9**

Table S6. Quality Assesment Scale—JBI checklist.

Criteria	Ben-Yakov	McAndrew	Vargo	Sami
**1. Patient demographics clearly described**	2	2	2	2
**2. Clinical history detailed chronologically**	2	2	2	2
**3. Diagnostic methods clearly reported**	2	2	2	2
**4. Intervention clearly described**	2	2	2	2
**5. Condition before/after intervention stated**	2	2	2	2
**6. Adverse events reported**	2	2	2	2
**7. Key takeaway message / clinical implication**	2	2	2	2
**Total Score**	**14/14**	**14/14**	**14/14**	**14/14**
